# Inhibition of ATL development in humanized mouse model by AZT/INF treatment

**DOI:** 10.1186/1742-4690-11-S1-P43

**Published:** 2014-01-07

**Authors:** Kenta Tezuka, Mami Tei, Takaharu Ueno, Runze Xun, Hidekatu Iha, Jun-ichi Fujisawa

**Affiliations:** 1 Department of Microbiology, Kansai Medical University, Hirakata, Osaka, Japan; 2 Department of Infectious Diseases, Faculty of Medicine, Oita University, Oita, Japan

## 

HTLV-1 infection of humanized NOG mice has been demonstrated to recapitulate the development of ATL-like symptoms within several months of infection. Infected human T-cells in these mice start to proliferate vigorously in a couple weeks after infection and the mice die of ALT-like lymphoproliferative disorder. Thus, this mouse model should provide a potent tool to analyze the *in vivo* effect of various candidates for ATL treatment.

Treatment of ATL with the combination of anti-viral agents, zidovudine (AZT) and interferon-alpha (IFN), has been reported to be highly effective, especially to indolent type, but the mechanism of action is totally unknown. We, therefore, examined the efficacy and the *in vivo* mechanism of AZT/IFN treatment in the humanized mouse system.

HTLV-1 infected humanized mice were inoculated daily with AZT and IFN from two to four weeks post infection and the number of infected cells and proviral loads (PVL) were analyzed. Treatment with either AZT or IFN alone attenuated the onset of lymphoproliferative disorder, whereas the combined treatment suppressed the growth of infected T-cells in PBL almost completely and the PVL remained low throughout lifetime. The suppressive effect is infected-cell specific because the number of uninfected human lymphocytes in PBL stayed constant on the administration of drugs.

It is suggested that infected cells expressing higher level of viral gene, most provably Tax, should have been selectively eliminated, since a similar suppressive effect has been obtained in HTLV-1 infected humanized mice treated with an Hsp90 inhibitor, 17-DMAG, which enhances the degradation of Tax.

